# Enhanced Risk for Epidemic Cholera Transmission, Haiti

**DOI:** 10.3201/eid3112.251157

**Published:** 2025-12

**Authors:** Andrew J. Curtis, V. Madsen Beau De Rochars, Melody Achille, Rigan Louis, Jayakrishnan Ajayakumar, Jacques Boncy, Afsar Ali, J. Glenn Morris

**Affiliations:** Case Western Reserve University School of Medicine, Cleveland, Ohio, USA (A.J. Curtis); Case Western Reserve University, Cleveland (. Ajayakumar); University of Florida, Gainesville, Florida, USA (V. Madsen Beau De Rochars, R. Louis, A. Ali, J.G. Morris Jr.); Université Quisqueya, Port-au-Prince, Haiti (M. Achille); Ministère de la Santé Publique et de la Population, Port-au-Prince (J. Boncy)

**Keywords:** cholera, bacteria, enteric infections, epidemic, Haiti, Vibrio cholerae

## Abstract

Sporadic cholera outbreaks continue to occur in Haiti. We used a novel space-time analysis to gain insight from limited government surveillance data. We identified concerning patterns of disease spread in areas known to be at high risk for epidemic cholera in and around the capital city of Port-au-Prince.

The potential for a major cholera outbreak in Haiti should concern countries of the Americas, particularly at a time when political turmoil has encouraged emigration from Haiti. Cholera was introduced into Haiti by United Nation peacekeepers in October 2010 (*1*). The resulting epidemic lasted until 2019, killing ≈10,000 persons and sickening >820,000 persons. In September 2022, a new cholera outbreak occurred, in which illness was caused by strains matching previously isolated environmental toxigenic *Vibrio cholerae* O1 strains from the Jacmel region south of the capital city of Port au Prince (PaP) (*2*,*3*). By the end of 2023, the resulting epidemic had caused ≈80,000 clinically diagnosed cases (*4*–*6*). Many of the affected neighborhoods along the coast in and proximate to PaP swelled because of migration by persons displaced by the 2010 earthquake. Those areas have informal settlement characteristics, including poorly constructed and densely packed residential areas, a lack of access to safe water, inadequate drainage, and poor sanitation. Adding further complexity, most of those areas are now controlled by violent gangs, which has resulted in an estimated 130,000 additional persons being displaced since the beginning of 2025 (*7*). Taken together, those factors have created a complex infectious disease vulnerability.

For the outside world, acquiring the data needed to understand the current cholera situation in Haiti is extremely difficult. Starting in June 2024, Haiti’s Ministere de La Publique et de La Population began releasing data on its website, including numbers of suspected cholera cases by commune (*4*,*5*). Those data, although likely to reflect a profound undercount (especially in areas controlled by gangs), can be used to track the rise and spread of cholera in and around PaP. We describe a methodology that is simple to implement, can easily be updated to provide a near real-time assessment, and can be adopted to monitor disease outbreaks in other countries.

A grid heat map, an exploratory space-time epidemic surveillance tool, is an alternative to traditional geographic information systems (GIS) mapping. Our grid includes all suspect cholera cases by commune for each week of the epidemic. As further weekly data become available, the grid recalculates to update the pattern. A neighborhood version of this grid heat map had previously been developed for Haiti during the 2023 epidemic (*6*). For 2024–2025, we visualized cholera in each commune by using the darkest shading for the week with most cases, then coloring all other weeks as a proportion of that maximum disease count. If the next week had even higher cases for that commune, the previous weeks are recalculated as a proportion of the new total. The finished heat map, which conceptually displays the epidemic curve for each commune, can reveal regional patterns that might be washed out using typical GIS approaches. 

We constructed a combined grid and cartographic heatmap interface programmed in Python (https://www.python.org) into which each weekly disease sheet is ingested when available (Appendix). We also extracted key maps from the grid heatmap coinciding with the rise in cases around PaP in the spring and summer of 2025 (Figure). 

**Figure:**
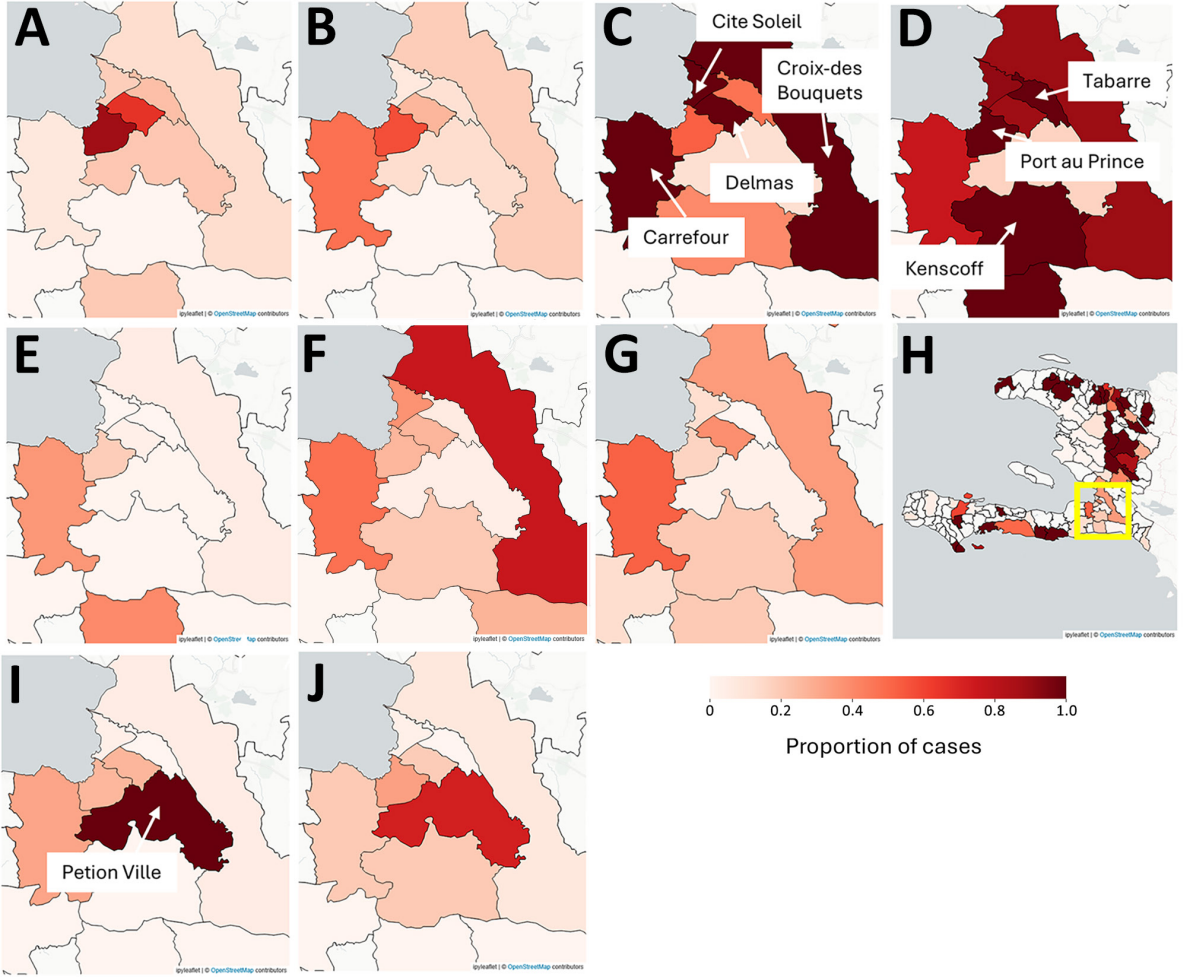
Maps showing enhanced risk for epidemic cholera transmission, Haiti. Extracted cartography are from the grid heat map disease monitoring system for Port-au-Prince during the summer surge of suspected cholera cases between the weeks ending April 20–October 13, 2025. A) April 20; B), April 27; C) May 14; D) June 1; E) June 20; F) June 29; G) August 20; H) August 20; I) October 7; J) October 13. The mapped area is identified on the August 20 map (panel H; yellow outline) for the whole country, which also shows the broader geographic spread of suspected cholera for that reporting period. The shading should be interpreted carefully because each commune is mapped according to where it falls along its own epidemiologic curve for that week. Each commune also can be compared to its neighbors in the same week by where they also fall along their curves, given that communes with the darkest shade are at their peak number of cases. The maps should not be interpreted as visualizing the actual number of cases per commune per week using the same classification scheme as one would in a typical cartographic display. Maps created by using OpenStreetMap (https://www.openstreetmap.org).

In early 2025, PaP and its vicinity started to see a concerning rise in cases (Figure; Appendix Figure). An earlier version of the grid heatmap in April 2025 captured the emergence of that signal. As cases rose, crucial weeks were those ending April 20, May 14, and June 1, when each of the communes in and around PaP had their highest total case counts. Although Cité Soleil, one of the most vulnerable communes within PaP, registered the highest number of cases (week ending April 20, 73 cases; May 14, 393 cases; June 1, 359 cases, and June 29, 145 cases), reflecting a similar pattern seen in the 2023 epidemic (*8*), traditional GIS mapping would have missed the situation in the less dense communes of Kenscoff (week ending June 1, 5 cases) and Tabarre (week ending June 1, 17 cases). Those maps suggest that although conditions in the coastal neighborhoods might be driving cholera amplification, epidemic spread beyond the city and into the surrounding communes is evident. That pattern carried into October, when Petion-Ville, south of PaP, saw some of its highest case numbers. We also observed a rise of intensity to the north, which matched geographic patterns previously seen in summer 2024 (Appendix Figure). The communes in the west of Haiti also saw some of their highest case numbers during the same week.

We believe those patterns reveal the widespread cholera vulnerability across Haiti. Although in summer 2025 we were concerned that the situation around PaP might have been primed for another sizeable cholera outbreak, by December 2025 that had not occurred, but the area in and around PaP continues to generate cases, especially in Petion-Ville commune. Even if the data are incomplete or suffer from inconsistent updates, they can still provide vital early warning signals. We continue to use this method to monitor changes in the cholera situation in Haiti. Available data underscore the critical need for near real-time analysis of surveillance data for countries like Haiti. 

Appendix Additional information about enhanced risk for epidemic cholera transmission, Haiti.
