# Supplementary material for: Enhanced Risk for Epidemic Cholera Transmission, Haiti
Source: Emerg Infect Dis. 2025 Dec;31(12):2329–32. doi: 10.3201/eid3112.251157 (PMC12782238; doi:10.3201/eid3112.251157)
Supplement: Appendix — Additional information about enhanced risk for epidemic cholera transmission, Haiti. [file 25-1157-Techapp-s1.pdf]

Article DOI: https://doi.org/10.3201/eid3112.251157

EID cannot ensure accessibility for supplementary materials supplied by authors. Readers who have difficulty accessing supplementary content should contact the authors for assistance.

Enhanced Risk for Epidemic Cholera Transmission, Haiti

Appendix

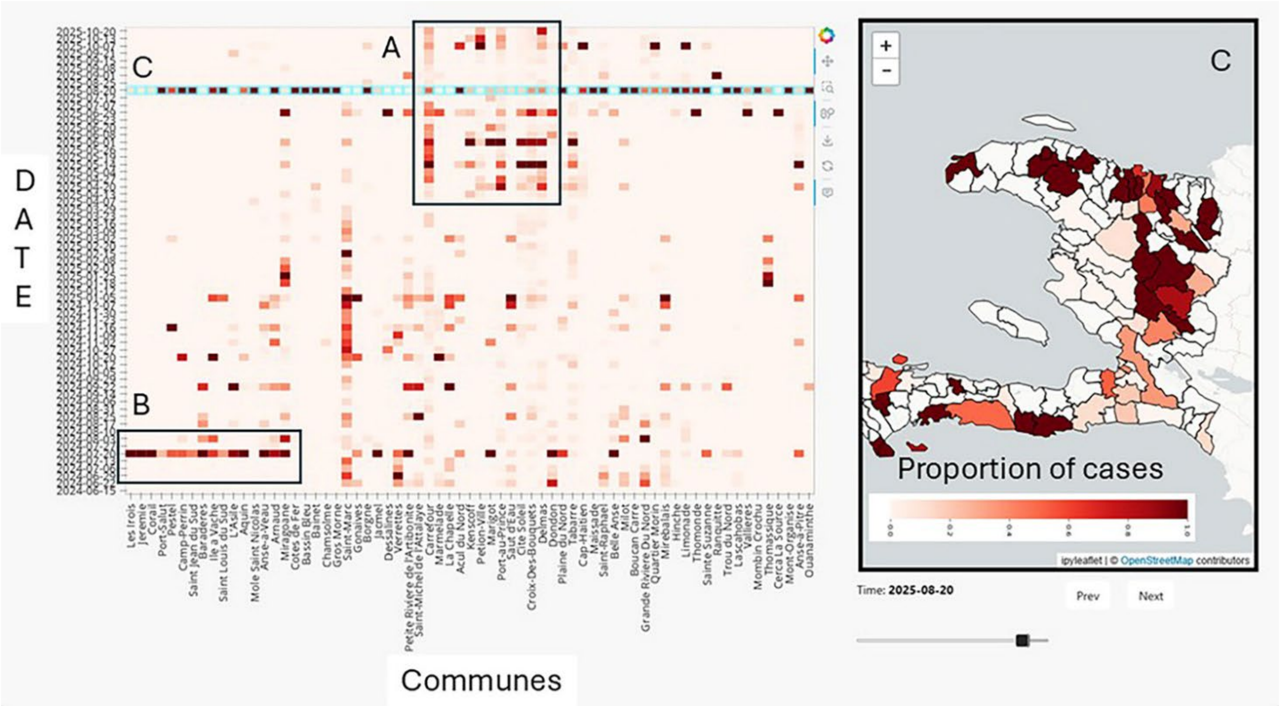

Appendix Figure. Static image taken from the dynamic grid heat map monitoring system designed to display suspected cholera cases for all communes in Haiti. Data periods occur during June 15, 2024–October 20, 2025. The ordering of communes can be sorted either alphabetically or geographically. Here they are sorted in an east-west direction to better visualize regional trends. Box A includes communes which mostly surround Port-au-Prince, including the areas and time frame mapped in Figure (https://wwwnc.cdc.gov/EID/article/31/12/25-1157-F1.htm). Box B displays communes with their highest case totals occurring in 2024, which were the same areas impacted during the 2010 epidemic, including Mirebalais where the originating UN camp was located. Interestingly some of the same communes reemerge in the highlighted week (indicated by C) and its associated map during August.
